# Attentiveness of pediatricians to primary immunodeficiency disorders

**DOI:** 10.1186/1756-0500-5-393

**Published:** 2012-07-31

**Authors:** Suleiman Al-Hammadi, Eiman Al-Reyami, Sareea Al-Remeithi, Khawla Al-Zaabi, Rola Al-Zir, Heba Al-Sagban, Taoufik Zoubaidi, Abdul-Kader Souid

**Affiliations:** 1 Department of Pediatrics, Faculty of Medicine and Health Sciences, UAE University, P.O. Box 17666, Al-Ain, United Arab Emirates; 2 Department of Pediatrics, Saqr Hospital, Ras Al-Khimah, United Arab Emirates; 3 Department of Pediatrics, Sheikh Khalifa Medical City, Abu Dhabi, United Arab Emirates; 4 Department of Pediatrics, Kalba Hospital, Sharjah, United Arab Emirates; 5 Department of Pediatrics, Al-Wasl Hospital, Dubai, United Arab Emirates; 6 Department of Pediatrics, Dubai Hospital, Dubai, United Arab Emirates; 7 Department of Statistics, Faculty of Business and Economics, UAE University, Al-Ain, United Arab Emirates

**Keywords:** Survey, Primary immunodeficiency, Knowledge, Diagnosis, Management

## Abstract

**Background:**

Primary immunodeficiency (PID) is a cluster of serious disorders that requires special alertness on the part of the medical staff for prompt diagnosis and management of the patient. This study explored PID knowledge and experience among pediatricians of wide educational backgrounds, practicing in the United Arab Emirates (UAE).

**Method:**

A self-administered questionnaire was used to determine the competency of pediatricians in their knowledge of PID disorders. This study questionnaire included questions on PID signs and symptoms, syndromes associated with immunodeficiency, screening tests, interpreting laboratory tests and case management. The participants were 263 pediatricians of diverse education working in the 27 governmental hospitals in all regions of UAE.

**Results:**

The overall performance of the pediatricians did not differ based on their age, gender, origin of certification, rank, or years of experience. Of the 50 questions, 20% of pediatricians answered correctly <60% of the questions, 76% answered correctly 60 to 79% of the questions, and 4% answered correctly ≥80% of the questions. Seventeen of the 19 PID signs and symptoms were identified by 55 to 97%. Four of 5 syndromes associated with immunodeficiency were identified by 50 to 90%. Appropriate screening tests were chosen by 64 to 96%. Attention to the laboratory reference range values as function of patient age was notably limited.

**Conclusions:**

There was a noteworthy deficiency in PID work-up. Therefore, implementing effective educational strategies is needed to improve the competency of pediatricians to diagnose and manage PID disorders.

## Background

Primary immunodeficiency (PID) is a cluster of disorders that share a common theme of excessive susceptibility to infection and other associated clinical problems [1,2]. These serious episodes of infection markedly impair the patients’ ability to lead a normal life [3,4]. While individual diseases are rare, PID as an entity is not uncommon [1]. To date, there are more than 160 genetically distinct PID diseases [5], affecting more than 10 million children and adults worldwide [6]. As appropriately stated, PID is common enough that a primary care physician is likely to encounter several patients in their practice [7]. Wood et al. indicated that diagnostic delays remain common, mostly due to limited awareness of the heterogeneity and presenting features of PID [8].

Familiarity of pediatricians with PID is essential for the early diagnosis and case management. Inadequate clinical and laboratory assessments account for most of the deferred diagnoses that may result in organ impairments [1,2]. A recent study suggested the awareness initiatives and educational programs should especially target pediatricians, hospitalists and families with members who have PID [9].

Historically, medical surveys addressing PID disorders have served various clinical needs, including identifying educational gaps. The United Arab Emirates (UAE) are especially distinguished by the diversity of medical education backgrounds of their pediatricians, which makes educational questionnaires especially meaningful and consequential. Moreover, parental consanguinities are typical in UAE, accounting for the possibility of a high occurrence of PID. However, since there is no national registry of PID in UAE, the prevalence of PID is unknown. For similar cultures, parental consanguinity among PID patients was found to be ~77% in Kuwait [10], ~66% in Iran [11] and ~63% in Egypt [12].

The primary objective of this study was to explore PID knowledge and experience among pediatricians of diverse educational backgrounds and practicing environments. The other objective was to identify educational gaps that needed to be overcome to improve the care of children with PID.

## Methods

A self-reported questionnaire was used to explore PID experience among pediatricians in UAE. The feasibility was assessed initially in the first 10 participating pediatricians. The questions were clear and fully answered.

Sixty-three items were included in the survey. Six questions looked at personal information, 24 at PID signs and symptoms, 9 at associated syndromes, 11 at screening tests, 6 at laboratory interpretations and 7 at management. The implemented questions were a modification of those developed by Al-Herz et al. [13].

There were 27 government-based hospitals in UAE; these centers provided community and tertiary services across the country. All these medical facilities were included in the survey. The total number of pediatricians working in these hospitals was 422; 263 (62.3%) pediatricians of them were recruited and participated in the study.

Data were analyzed using SPSS statistical package (version 19). Poisson regression models were used to test for significant differences in the score (correct answers) in PID knowledge and investigation. The variables included pediatricians’ age, gender, certification, rank and years of experience.

The Poisson regression models fitted the data well; the *p*-values of Likelihood Ratio tests for goodness-of-fit were all > 0.90. Comparison of the median relative scores (percentage of correct answers) in PID knowledge and investigation were done using the Wilcoxon signed rank test. The same test was also used to obtain confidence intervals for the median relative scores in various components of the questionnaire (signs and symptoms, syndromes associated with immunodeficiency, screening tests and laboratory interpretation). The Wilcoxon nonparametric test was used because neither the relative scores nor their arcsine transformations (angular transformation) were normally distributed (Shapiro-Wilk test *p* < 0.001). The chi-square test for goodness-of-fit was used to compare the frequency of responses in each of the following variables: IVIG dosing, dosing intervals, and prophylactic antibiotic choices. A *p* ≤0.05 was considered significant.

The study was approved by the Ethics Committee of the Faculty of Medicine and Health Sciences at UAE University (No. 09/51).

## Results

Two hundred sixty three pediatricians (59% male) participated in the study. Their age (mean ± SD) was 42.1 ± 9.6 years.

Twenty-two percent had FRCP (or MRCP), 13% Pediatric Arab Board, 12% Diploma of Child Health, 11% Pediatric American or Canadian Board, and 41% other certifications (e.g., FACHARTZ and Master Degrees). Fifty percent were Specialists, 26% were house officers (or pediatric residents) and 24% were consultants. Forty-one percent practiced pediatrics <10 years, 40% 10 to 20 years, and 19% >20 years (mean ± SD = 13.4 ± 9.0 years; range = 1 to 40 years).

Table 1 shows the frequency of recognizing *common* PID signs and symptoms. Nineteen items pointed to PID signs and symptoms and 5 items (labeled with asterisks) were not. These manifestations were chosen to cover the four main components of the immune system, i.e., antibody deficiency, T-cells defect, neutrophil defect and complement deficiency. Five of the "ten warning signs" of PID [6] were included in the list (36% of participants answered the 5 questions correctly, 31% answered 4 questions correctly and 33% answered 1-3 questions correctly). Seventeen of the 19 PID signs and symptoms were frequently (55 to 97%) selected. Polyendocrinopathy and transfusion reaction were the least selected (48% and 44%, respectively). The five distractants were much less frequently selected.

**Table 1:** Which of the following signs and symptoms make you suspect PID?

	***Percent answered “yes”***
Persistent oral thrush	*97*
Infections with uncommon organisms	*96*
Failure of infant to thrive	*90*
Sepsis with atypical mycobacteria	*83*
Severe dermatitis	*81*
Two invasive deep seated infections	*80*
Delayed wound healing	*79*
Death of a sibling in infancy	*77*
Absent tonsils	*75*
Autoimmune cytopenia	*65*
Partial albinism	*65*
Lymphoma	*64*
Otitis media (5 bouts per year)	*62*
Interrupted aortic arch	*62*
Pneumonia (2 bouts per year)	*59*
Severe warts	*57*
Severe periodontitis	*55*
Polyendocrinopathy	*48*
Dextrocardia *	*45*
Transfusion reaction	*44*
Malar rash *	*28*
Inguinal lymphadenopathy *	*22*
Polydactaly *	*20*
Ventricular septal defect *	*9*

*Does not increase likelihood of PID.

(The order of the items is presented according to the percentage picked by respondents, not how it appeared in the questionnaire).

Table 2 shows the frequency of recognizing syndromes or conditions that may associate with PID [14]. Similarly, 4 of 5 syndromes associated with immunodeficiency were frequently (50 to 90%) selected. The four distractants (labeled with asterisks) were much less (≤20%) selected. The results in Tables 1,2 show PID knowledge is not limited in this cluster of pediatricians, despite their broad educational diversity.

**Table 2:** Which of the following syndromes or conditions may associate with PID?

	***Percent answered “yes”***
Ataxia telangiectasia	*90*
Bloom syndrome	*67*
Cartilage hair hypoplasia	*58*
Ectodermal dysplasia	*50*
Short-limb dwarfism	*33*
Ehler Danlos syndrome *	*20*
Hurler syndrome *	*18*
Turner syndrome *	*13*
Sturge Weber syndrome *	*9*

* Not associated with PID.

(The order of the items is presented according to the percentage picked by respondents, not how it appeared in the questionnaire).

Table 3 shows the frequency of selecting appropriate PID screening tests. Four items (complete blood counts with differential, quantitative serum immunoglobulins, lymphocyte subsets and chest x-ray) are stressed to be the initial screening before the next level of evaluation [2]. The remaining items (labeled with asterisks) were either next level of investigation (antibody titers to previous vaccines, lymphocyte stimulation, neutrophil oxidative burst, IgG subclasses and total complements), not commonly used (serum isohemagglutinins) or inappropriate (chest CT scan) [15]. The four appropriate screening tests were highly selected (64 to 96%). Unnecessary tests were frequently requested for screening, and selecting appropriate initial work-up of PID was imprecise. For example, IgG subclasses were selected as a screening test in 77% and neutrophil oxidative burst in 66%.

**Table 3:** Which of the following tests would you request for PID initial screening?

	***Percent answered “yes”***
CBC with differential	*96*
Serum immunoglobulin levels	*96*
IgG subclasses *	*77*
Chest x-ray	*76*
Neutrophil oxidative burst assay*	*66*
Antibody titers to previous vaccines *	*64*
Lymphocyte subsets	*64*
Total complements*	*56*
Lymphocyte stimulation tests *	*44*
Serum isohemagglutinins *	*40*
Chest CT scan *	*26*

* Not recommended as a first-line screening.

(The order of the items is presented according to the percentage picked by respondents, not how it appeared in the questionnaire).

Table 4 included six laboratory findings; three of them were age-dependent. Absent delayed hypersensitivity skin reaction to Candida is only abnormal after 12 months of age [16]; this item was incorrectly selected by 74%. The absolute lymphocyte count at 2 to 5 months of age should be > 3,700/μL; thereafter, it decreases gradually to > 1,700/μL by 2 years of age [17,18]. This question was correctly answered by 54%. A serum IgG level of 350 mg/dL is acceptable up to one year of age; followed by a gradual increase until 6 years of age (≥ 600 mg/dL) [15]; this item was incorrectly selected by 35%. These results suggest that attentiveness to normal laboratory values as function of age is limited.

**Table 4:** Which of the following results suggest PID?

	***Percent answered “yes”***
Absent delayed hypersensitivity skin reaction to Candida in a 6-month-old infant *	*74*
Giant granules in neutrophils	*64*
Absolute lymphocyte count < 2,500/μL in a 4-month-old infant	*54*
Small size platelets	*44*
Serum IgG level 350 mg/dL in a 7-month-old infant *	*35*
Neonatal thrombocytopenia	*31*

* Not a finding that suggests PID.

(The order of the items is presented according to the percentage picked by respondents, not how it appeared in the questionnaire).

**Figure 1:**
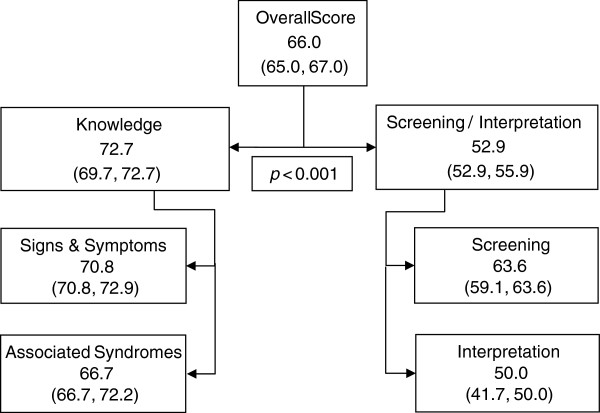
Median percent scores (95% confidence intervals for median percent scores) per categories.

Table 5 and Figure 1 show the overall performance scores (not including the management questions) and classifying the sum of correct answers to three levels of competency: ≥80%, 60 to 79% and <60%. The majority (76%) of pediatricians correctly answered 60 to 79% of the questions, and only 4% correctly answered ≥80% of the questions. Significant differences were noted between the two main categories (*p*-value < 0.001, Wilcoxon signed rank test), with a better knowledge score (95% CI: 69.7, 72.7) than investigation/interpretation score (95% CI: 52.9, 55.9).

**Table 5:** The performance scores

Performance score*	**Knowledge**^**†**^	**Investigation**^**†**^	**Overall**^**†**^
	**(Tables**1 **,**2**)**	**(Tables**3 **,**4**)**	**(Tables**1 **,**2 **,**3 **,**4**)**
<60	15%	54%	20%
60 to 79%	63%	44%	76%
≥ 80%	22%	2%	4%

* Percent of correct answers.

^†^ Percent of pediatricians scored in each category.

The overall performance scores did not significantly differ with age (Poisson regression, likelihood ratio test *p* = 0.736), gender (*p* = 0.382), certification (*p* = 0.435), rank (*p* = 0.829) and experience (*p* = 0.706). Similarly, the knowledge and investigation/interpretation scores (when assessed separately) did not differ with age, gender, certification, rank and experience (all *p* > 0.20).

Intravenous immunoglobulin dosing and administration intervals were also tested. Sixty-five percent choose a dose of 400 to 600 mg/kg and 80% choose 3 to 4 week dosing intervals [1]. Eight percent did not consider mg/kg as a basis for dosing. For prophylactic antibiotics, 44% recommended co-trimoxazole, 21% recommended penicillins and 23% recommend none.

Overall, 47% had at least a patient with PID, 41% had referred a patient for PID assessment, and 12% had neither evaluated nor referred a patient with PID. Only 9% of the pediatricians were completely comfortable with PID patients, 59% were somewhat comfortable, and 32% were not comfortable.

## Discussion

The most important finding of this study was the fact that most pediatricians (despite their diverse education, ranks and years of experience) recognized the common signs and symptoms of PID. There were significant problems, however, in requesting the appropriate tests, identifying the age-appropriate reference values and interpreting the results. Although the theoretical knowledge is reasonable, these data show limitations in practicing for children suspected to have PID. It worth noting that despite the study aimed primarily to assess knowledge in practicing pediatricians, 26% of the participants were house officers or pediatric residents. Interpreting laboratory results should discern normal values for age, as the first few years reveal enormous immune maturation [2].

PID knowledge and practice have been explored in a limited number of studies. In a study from Kuwait, 26% of the pediatricians correctly answered ≥ 67% of the questions. The mean overall score was 60% (95% confidence interval = 58% to 61%), the clinical presentation score was 63%, the syndromes associated with immunodeficiency score was 58%, and the laboratory investigation score was 51%. It was uncertain, however, whether these results are applicable to other countries and repeated studies are recommended [13]. Another study from Turkey investigated only the awareness of pediatricians to important PID indicators. Family history was identified by 91%, persistent thrush by 90%, consanguineous marriages by 87%, telangiectasia by 82%, failure to thrive by 79%, neonatal tetany by 78%, absence of tonsils by 75%, oculocutaneous albinism by 74%, hospitalization for recurrent infection by 71%, resistant sinusitis by 71%, infant deaths by 70%, giardiasis by 62%, liver abscess by 61%, recurrent oral aphthous by 59% and poliomyelitis following oral polio vaccination by 51% [19].

Early diagnosis of PID is essential to avoid serious sequelae. Comprehensive immune work-up that includes genetic analysis is not readily available in many parts of the world. Nevertheless, this short coming was usually not the reason for the delayed diagnosis. It has been shown that hindrance in PID diagnosis hinges mainly on proper consideration of the clinical and laboratory findings [20].

The cornerstones for PID diagnosis are medical history (focused on recognizing PID symptoms), physical examination (focused on indicative PID signs) and laboratory investigation (focused on pathognomonic recognition of the anomalies). These foundations need to be implemented as a practical diagnostic strategy.

Proper patient management should promptly follow the diagnosis. Immunoglobulin and antimicrobial therapies are usually available. Allogeneic hematopoietic stem cell transplantation, on the other hand, requires referral to a tertiary center. Other modalities include gene (e.g., severe combined immune deficiency and chronic granulomatous disease), enzyme (e.g., adenosine deaminase deficiency) and immune modulation (e.g., immune dysregulation) therapies [1].

Establishing a national registry is a critical need and would serve numerous purposes. It would provide epidemiological data, and address ethnical and geographical variations [10]. Rezaei et al. documented improved recognition of PID disorders following the creation of PID registries [20]. Simplifying the educational materials and provision of interactive learning appear to be more effective than conventional Continuous Medical Education courses. Training strategies should include understanding the development of the immune system, comprehending the complexity of the investigations and interpreting the results appropriately.

## Conclusions

The clinical gaps reported here likely stem from inadequate engagements of pediatricians in the work-up of children with PID. The usual practice is to refer these patients to immunologists without effective investigation. Moreover, the communication between immunologists and pediatricians typically ceases at patient referral. This practice is further enforced by fears involved in caring for these vulnerable patients. Since these disorders are relatively rare, opportunities to be familiar with them are relatively infrequent.

The clinical practice needs to allow pediatricians to take initiatives and provide care for PID patients, especially with limited number of immunologists worldwide. Immunologists should encourage pediatricians’ involvement in the patient care and make themselves available for consultations. The exchanges between immunologists and pediatricians should be informative and educational. Pediatricians, on the other hand, may need to concentrate more on pattern recognition, work-up and laboratory interpretation rather than molecular diagnosis.

## Authors’ contributions

SAH and AKS have made substantial contributions to conception and design, acquisition of data, and analysis and interpretation of data. They also have been involved in drafting the manuscript and revising it critically for important intellectual content. EAR, SAR, KAZ, RAZ and HAS have made substantial contributions to acquisition of data, and have given their final approval of the version to be published. TZ have made substantial contributions to analysis and interpretation of data, and have given final approval of the version to be published. All authors read and approved the final manuscript.

## Competing interests

The authors declare that they have no competing interests.

## Authors’ information

SAH is Clinical Immunologist in the Faculty of Medicine and Health Sciences (FMHS) - UAE University. AKS is Chairman of the Department of Pediatrics (FMHS - UAE University). TZ is biostatistician (UAE University). EAR, SAR, KAZ, RAZ and HAS are pediatricians practicing in the UAE.

## References

[B1] Ochs HD Stiehm R Winkelstein J Ochs HD, Stiehm R, Winkelstein J Antibody deficiencies Immunologic disorders in infants & children 2004 5 Elsevier Saunders, Philadelphia 356 426

[B2] Leung DYM Sampson HA Geha R Szefler SJ Pediatric Allergy Principles and Practice 2010 2 Elsevier Saunders, Philadelphia

[B3] Nicolay U Kiessling P Berger M Gupta S Yel L Roifman CM Gardulf A Eichmann F Haag S Massion C Ochs HD Health-related quality of life and treatment satisfaction in North American patients with primary immunodeficiency diseases receiving subcutaneous IgG self-infusion at home J Clin Immunol 2006 26 1 65 72 10.1007/s10875-006-8905-x 16418804

[B4] Aghamohammadi A Montazeri A Abolhassani H Saroukhani S Pourjabbar S Tavassoli M Darabi B Imanzadeh A Parvaneh N Rezaei N Health-related quality of life in primary antibody deficiency Iran J Allergy Asthma Immunol 2011 10 1 47 51 21358015

[B5] Al-Herz W Bousfiha A Casanova JL Chapel H Conley ME Cunningham-Rundles C Etzioni A Fischer A Franco JL Geha RS Hammarström L Nonoyama S Notarangelo LD Ochs HD Puck JM Roifman CM Seger R Tang MLK Primary Immunodeficiency Diseases: an update on the Classification from the International Union of Immunological Societies Expert Committee for Primary Immunodeficiency Front Immunol 2011 2 1 26 22566844 10.3389/fimmu.2011.00054 PMC3342372

[B6] Jeffrey Modell foundation website[http://www.jmfworld.com] (August 30, 2011

[B7] Boyle JM Buckley RH Population prevalence of diagnosed primary immunodeficiency diseases in the United States J Clin Immunol 2007 27 5 497 502 10.1007/s10875-007-9103-1 17577648

[B8] Wood P Herriot R Jones A Chapel H Burton J Stanworth S Peckham D Hyde C Hughan C (UK Primary Immunodeficiency Network). Primary antibody deficiencies: recognition, clinical diagnosis and referral of patients Clin Med 2009 9 6 595 599 20095309 10.7861/clinmedicine.9-6-595 PMC4952305

[B9] Subbarayan A Colarusso G Hughes SM Gennery AR Slatter M Cant AJ Arkwright PD Clinical features that identify children with primary immunodeficiency diseases Pediatrics 2011 127 5 810 816 10.1542/peds.2010-3680 21482601

[B10] Al-Herz W Primary immunodeficiency disorders in Kuwait: first report from Kuwait National Primary Immunodeficiency Registry (2004–2006) J Clin Immunol 2008 28 2 186 193 10.1007/s10875-007-9144-5 18008151 PMC7102084

[B11] Rezaei N Pourpak Z Aghamohammadi A Farhoudi A Movahedi M Gharagozlou M Mirsaeid Ghazi B Atarod L Abolmaali K Mahmoudi M Mansouri D Arshi S Tarash NJ Sherkat R Amin R Kashef S Hosseini RF Mohammadzadeh I Shabestari MS Nabavi M Moin M Consanguinity in primary immunodeficiency disorders; the report from Iranian Primary Immunodeficiency Registry Am J Reprod Immunol 2006 56 2 145 151 10.1111/j.1600-0897.2006.00409.x 16836617

[B12] Reda SM Afifi HM Amine MM Primary immunodeficiency diseases in Egyptian children: a single-center study J Clin Immunol 2009 29 3 343 351 10.1007/s10875-008-9260-x 19002574

[B13] Al-Herz W Zainal ME Salama M Al-Ateeqi W Husain K Abdul-Rasoul M Al-Mutairi B Badawi M Aker N Kumar S Al-Khayat H Primary immunodeficiency disorders: survey of pediatricians in Kuwait J Clin Immunol 2008 28 4 379 383 10.1007/s10875-008-9191-6 18351445

[B14] Kersseboom R Brooks A Weemaes C Educational paper: syndromic forms of primary immunodeficiency Eur J Pediatr 2011 170 3 295 308 10.1007/s00431-011-1396-7 21337117 PMC3068525

[B15] Bonilla F Leung DYM, Sampson HA, Geha R, Szefler SJ Antibody Deficiency Pediatric Allergy Principle and Practice 2010 2 Elsevier Saunders, Philadelphia 88 97

[B16] Kniker WT Lesourd BM McBryde JL Corriel RN Cell-mediated immunity assessed by Multitest CMI skin testing in infants and preschool children Am J Dis Child 1985 139 8 840 845 4025264 10.1001/archpedi.1985.02140100102044

[B17] Appendix 1 Leung DYM, Sampson HA, Geha R, Szefler SJ Clinical Immunology Laboratory value Pediatric Allergy Principle and Practice 2010 2 Elsevier Saunders, Philadelphia 664 665

[B18] Adeli MM Buckley RH Why newborn screening for severe combined immunodeficiency is essential: a case report Pediatrics 2010 126 2 e465 e469 10.1542/peds.2009-3659 20603253

[B19] Yüksek M Ikincioğullari A Doğu F Elhan A Yüksek N Reisli I Babacan E Primary immune deficiency disease awareness among a group of Turkish physicians Turk J Pediatr 2010 52 4 372 377 21043382

[B20] Rezaei N Aghamohammadi A Moin M Pourpak Z Movahedi M Gharagozlou M Atarod L Ghazi BM Isaeian A Mahmoudi M Abolmaali K Mansouri D Arshi S Tarash NJ Sherkat R Akbari H Amin R Alborzi A Kashef S Farid R Mohammadzadeh I Shabestari MS Nabavi M Farhoudi A Frequency and clinical manifestations of patients with primary immunodeficiency disorders in Iran: update from the Iranian Primary Immunodeficiency Registry J Clin Immunol 2006 26 6 519 532 10.1007/s10875-006-9047-x 17024564

